# Effects of Changing the Reporting System from Decentralized/Modality-Based to Centralized/Subspecialized Radiology on Radiologists, Radiologic Technicians and Referring Physicians of a Multi-Center Radiology Network

**DOI:** 10.5334/jbsr.2483

**Published:** 2021-09-16

**Authors:** Andreas Zabel, Sebastian Leschka, Tim Fischer, Simon Wildermuth, Tobias Dietrich

**Affiliations:** 1 Kantonsspital Sankt Gallen, CH

**Keywords:** Specialization, Change Management, Process Assessment, Comparative Studies, Socioeconomic Issues

## Abstract

**Objectives:**

To determine the effects of reorganizing a radiology institute from decentralized/modality-based to centralized/subspecialized radiology on radiologists, radiologic technicians, and referring physicians at a multi-center radiology network.

**Material and Methods:**

In 2017/2018 our multi-center radiology network was changed from decentralized/modality-based to centralized/subspecialized reporting. A survey was conducted among radiologists, technicians and two groups of referring physicians (main hospital and non-main hospitals). The following items were tested: Overall satisfaction, perceived quality of radiological reports, subjective productivity/efficiency, confidence of radiologists in their subspecialty, availability of radiologists and turnaround time. Two of five answering options on a 5-point Likert scale were considered to represent agreement. The Mann-Whitney-U-test served for statistical analyses in agreement before and after reorganization in each group.

**Results:**

For radiologists, a significant difference was observed in perceived quality of radiological reports 42/46 (91.3%) compared to 51/52 (98.1%; p = 0.013).

For technicians, no significant differences were observed. In the group of main hospital referring physicians, significant differences were observed in overall satisfaction 129/152 (84.9%) compared to 164/174 (94.3%; p < 0.001) and in perceived quality of radiological reports 125/148 (72.8%) compared to 157/170 (92.4%; p = 0.001). In the group of non-main hospital referring physicians no significant differences were observed.

**Conclusion:**

The reorganization resulted in a significantly higher perceived quality of radiological reports for the groups of radiologists and main hospital referring physicians besides overall satisfaction for main hospital referring physicians. Specialized main hospital referring physicians value reports of specialized radiology, whereas less specialized, non-main hospital referring physicians did not experience any significant effect.

## Introduction

Our public radiology network consists of a main hospital and nine affiliated radiology locations at nine hospitals of different sizes between approximately 100 and 800 beds and one additional dedicated outpatient imaging center. The radiology network is an integrated imaging service for public primary, secondary, and tertiary health care service provider that covers approximately 3000 km^2^ and a population of approximately 1,000,000 inhabitants [1,2]. Originally each imaging center was independently organized with a modality-based reporting system. In January 2018 this decentralized/modality-based workflow was replaced by centralized and subspecialized, organ-based radiology [1]. Therefore, organ-based radiology teams were established, and most radiologists were transferred from the external locations to the main hospital [1].

We hypothesized that the reorganization from decentralized/modality-based to centralized/subspecialized radiology reporting has a positive effect on employees (radiologists, radiologic technicians) and referring physicians. The purpose of this study was to determine the effects of restructuring the reporting system from decentralized/modality-based to centralized/subspecialized radiology in overall satisfaction, perceived quality of radiological reports, subjective productivity/efficiency of employees, confidence of radiologists in their chosen subspecialty, availability of radiologists and turnaround time.

## Material and Methods

### Questionnaires

A total of four questionnaires were developed and distributed to the following groups:

– Radiologists [board-certified radiologists and radiology residents; (R)]
– Radiologic technicians (T)
– Referring physicians of the main hospital (H)
– Referring physicians of the non-main hospitals (N)

There were eight questions for radiologists, seven questions for radiologic technologists and five questions for each group of referring physicians (main hospital/non-main hospitals) (***Table 4***). Most of the questions were based on a 5-point Likert scale [3] (available responses: Agree Completely, Agree Somewhat, Neutral, Disagree Somewhat, Disagree Completely) as well as a non-applicable option [4]. In general, two subtypes of questions were distributed. Questions regarding one of both examined systems of radiological reporting were marked alphabetically (e.g., Ra). Comparative questions between both systems of radiological reporting were marked numerically (e.g., R1).

All groups were asked the following questions:

– Ra/Ta/Ha/Na: Overall, how satisfied have you been with decentralized/modality-based radiology reporting (before system change)?
– Rb/Tb/Hb/Nb: Overall, how satisfied have you been with centralized/subspecialized radiology reporting (after system change)?

The following questions were asked in the groups of radiologists (R), referring physicians of the main hospital (H), and referring physicians of the non-main hospitals (N):

– Rc/Hc/Nc: How did you perceive the quality of radiology reports with decentralized/modality-based radiology reporting (before the system change)?
– Rd/Hd/Nd: How did you perceive the quality of radiology reports with centralized/subspecialized radiology reporting (after system change)?

The following question was asked in the groups of radiologists (R), and radiologic technicians (T):

– R1/T1: How would you rate your subjective productivity/efficiency since changing from decentralized/modality-based to centralized/subspecialized radiology reporting (comparison between both evaluated systems of radiological reporting)?

Furthermore, group-specific questions were distributed:

#### Radiologists (R)

– R2: How did you perceive the quality of radiology reports since changing from decentralized/modality-based to centralized/subspecialized radiology reporting (comparison between both evaluated systems of radiological reporting)?
– R3: How professionally confident are you in your assigned subspecialization since changing from decentralized/modality-based to centralized/subspecialized radiology reporting (comparison between both evaluated systems of radiological reporting)?

#### Radiologic technicians (T)

– Tc: How would you rate the availability of residents (Radiology) with decentralized/modality-based radiology (before system change)?
– Td: How would you rate the availability of residents (Radiology) with decentralized/modality-based radiology (after system change)?
– Te: How would you rate the availability of senior-radiologists with decentralized/modality-based radiology (before system change)?
– Tf: How would you rate the availability of senior-radiologists with decentralized/modality-based radiology (after system change)?

#### Referring physicians of the main hospital (H), and referring physicians of the non-main hospitals (N)

– H1/N1: How would you rate the radiology turnaround time since changing from decentralized/modality-based to centralized/subspecialized radiology reporting (comparison between both evaluated systems of radiological reporting)?

**Figure 1:**
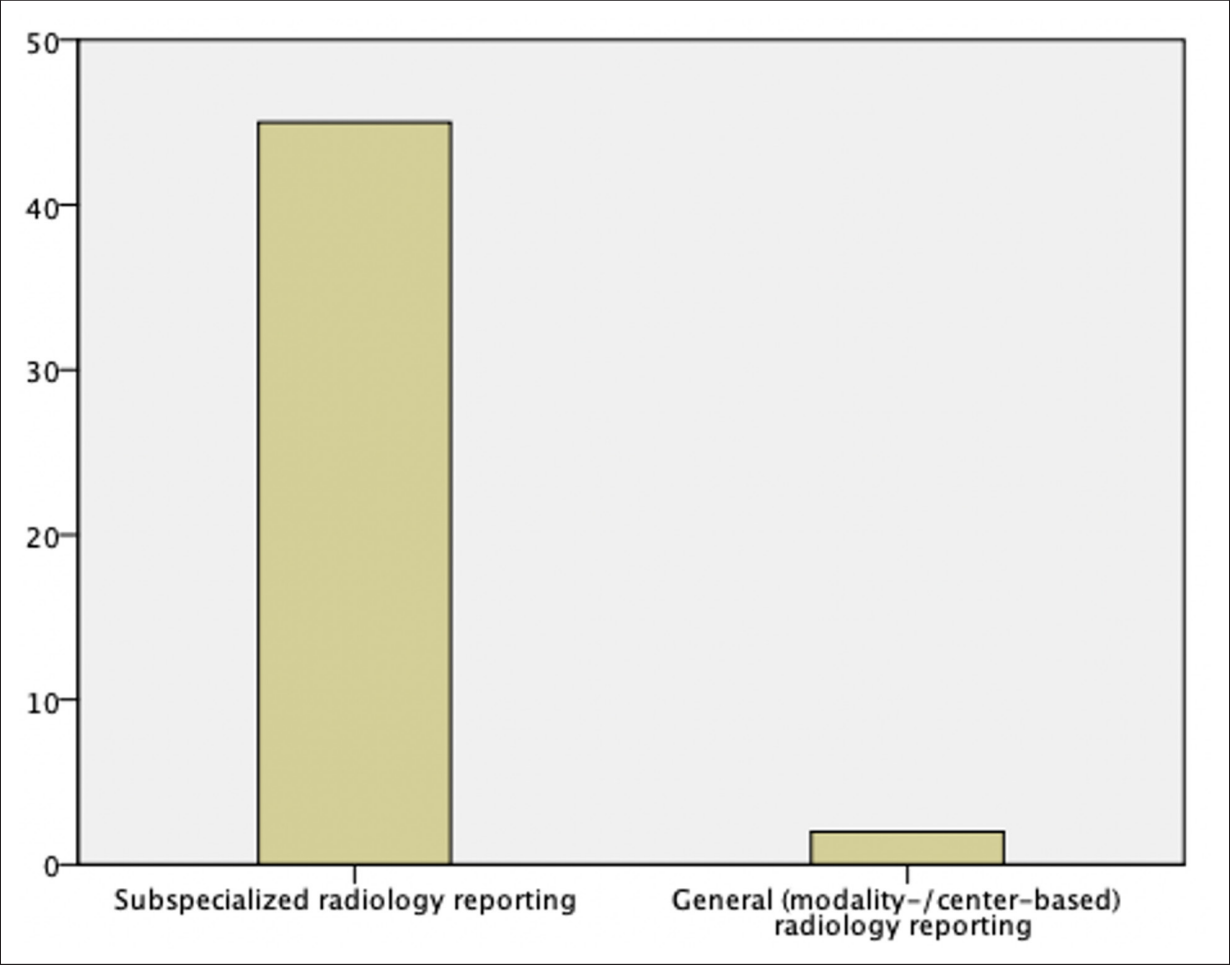
Distribution of responses to the question: “Which system of radiological reporting do you prefer?” This question was reserved for the group of radiologists (N = 55). Forty-seven radiologists answered the question. Forty-five radiologists (95.7%) preferred the centralized/subspecialized system of radiological reporting. Seven radiologists chose the non-applicable option (excluded). One missing answer (excluded).

In addition to the above-mentioned 5-item Likert scale questions, a question with three possible answers was included in the questionnaire for radiologists (***Figure 1***):

– Which system of radiological reporting do you prefer (available multiple-choice responses: decentralized/modality-based or centralized/subspecialized reporting or non-applicable)?

### Data acquisition

The reorganization of the institute from decentralized/modality-based to centralized/subspecialized radiology took place in January 2018. The interval from January 2018 to January 2019 was considered as a transitional one-year period, during which all four examined groups could get familiar with the centralized/subspecialized reporting system. The survey was open between January and May 2019. The survey data was collected via a commercial online platform (Surveymonkey, USA). The weblink to the internet-based survey was distributed through two invitation emails in January and April 2019. Participation in the survey was voluntary and anonymous. No individual responses were evaluated.

### Statistics

Answers on the 5-point Likert scale were grouped as follows: Answering options “Agree Completely” and “Agree Somewhat” were considered to represent agreement and were reported as absolute agreement as well as relative agreement in per cent (%). Questions, assessing the situation in one of both examined systems of radiological reporting (marked alphabetically) were compared to each other in each group. Questions answered with a non-applicable answering option were excluded from the analysis (***Table 1***). Statistical analyses were performed using SPSS (IBM Corporation, USA). The Mann-Whitney-U-test was used for statistical analyses in agreement before and after reorganization in each group. P values <0.05 were considered to denote statistically significant differences.

**Table 1:** Distribution of all responses listed in detail in Tables 2 and 3.


	N	%

Agree Completely	1,131	21.7

Agree Somewhat	1,907	36.5

Neutral	1,225	23.5

Disagree Somewhat	158	3.0

Disagree Completely	15	0.3

Non-applicable	764	14.6

Missing answers	18	0.3

Total	5,218	100


## Results

A total of 410 questionnaires from the groups of radiologists (56 questionnaires), radiologic technicians (78 questionnaires), referring physicians of the main hospital (178 questionnaires) and referring physicians of the non-main hospitals (98 questionnaires) were answered. The response rates were 65.6% for radiologic technicians and 96.6% for radiologists.

The agreement for overall satisfaction (questions Ra/b, Ta/b, Ha/b, Na/b; ***Tables 2, 3***) was high in both radiological reporting systems in all groups and ranged between 74,5% (Na) to 94,3% (Hb). In the group of referring physicians of the main hospital there was a significant (p < 0.001) difference in agreement for overall satisfaction of 129/152 (84.9%) for decentralized/modality-based radiology compared to 164/174 (94.3%) for centralized/subspecialized reporting (***Table 3***).

**Table 2:** Results of the questions for radiologists and radiologic technicians.


RADIOLOGISTS (R) AND RADIOLOGIC TECHNICIANS (T)							

QUESTIONS	N (%)					APPROVEMENT LEVEL	P-VALUE

	AGREE COMPLETELY	AGREE SOMEWHAT	NEUTRAL	DISAGREE SOMEWHAT	DISAGREE COMPLETELY		

**Ra**	22 (47.8)	17 (37.0)	7 (15.2)	0	0	84.8%	p = 0.911

**Rb**	27 (54.0)	13 (26.0)	5 (10.0)	5 (10.0)	0	80.0%	

**Ta**	19 (27.5)	28 (40.6)	17 (24.6)	5 (7.2)	0	68.1%	p = 0.158

**Tb**	28 (38.4)	27 (37.0)	15 (20.5)	2 (2.7)	1 (1.4)	75.5%	

**Rc**	9 (19.6)	33 (71.7)	4 (8.7)	0	0	91.3%	**p = 0.013**

**Rd**	21 (40.4)	30 (57.7)	1 (1.9)	0	0	98.1%	

**R1**	5 (10.6)	25 (53.2)	13 (27.7)	4 (8.5)	0	63.8%	Not applicable

**T1**	8 (11.8)	28 (41.2)	25 (36.8)	7 (10.3)	0	53.0%	Not applicable

**R2**	7 (14.6)	26 (54.2)	13 (27.1)	2 (4.2)	0	68.8%	Not applicable

**R3**	10 (22.7)	19 (43.2)	14 (31.8)	1 (2.3)	0	65.9%	Not applicable

**Tc**	10 (14.1)	47 (66.2)	11 (15.5)	2 (2.8)	1 (1.4)	80.3%	p = 0.87

**Td**	16 (21.6)	40 (54.1)	11 (14.9)	7 (9.5)	0	75.7%	

**Te**	11 (16.4)	36 (53.7)	17 (25.4)	3 (4.5)	0	70.1%	p = 0.277

**Tf**	15 (21.7)	39 (56.5)	11 (15.9)	4 (5.8)	0	78.2%	


[Capital-letters (‘R’ and ‘T’) indicate R = radiologists and T = radiologic technicians. Lower-case letters (‘a’, ‘b’, ‘c’, ‘d’, ‘e’ and ‘f’’) and the digits (‘1’, ‘2’ and ‘3’) abbreviate the individual questions as listed in Table 4].

**Table 3:** Results of the questions for referring physicians.


REFERRING CLINICIANS – MAIN HOSPITAL (H) AND NON-MAIN HOSPITALS (N)							

QUESTIONS	N (%)					APPROVEMENT LEVEL	P-VALUE

	AGREE COMPLETELY	AGREE SOMEWHAT	NEUTRAL	DISAGREE SOMEWHAT	DISAGREE COMPLETELY		

**Ha**	53 (34.9)	76 (50.0)	20 (13.2)	3 (2.0)	0	84.9%	**p < 0.001**

**Hb**	99 (56.9)	65 (37.4)	9 (5.2)	1 (0.6)	0	94.3%	

**Na**	30 (44.1)	21 (30.9)	15 (22.1)	2 (2.9)	0	74.5%	p = 0.951

**Nb**	40 (44.0)	31 (34.1)	14 (15.4)	5 (5.5)	1 (1.1)	78.1%	

**Hc**	23 (15.5)	102 (57.3)	22 (14.9)	1 (0.7)	0	72.8%	**p = 0.001**

**Hd**	51 (30.0)	106 (62.4)	13 (7.6)	0	0	92.4%	

**Nc**	13 (19.4)	36 (53.7)	16 (23.9)	2 (3.0)	0	73.1%	p = 0.677

**Nd**	15 (16.1)	59 (63.4)	19 (20.4)	0	0	79.5%	

**H1**	38 (26.4)	66 (45.8)	39 (27.1)	1 (0.7)	0	72.2%	Not applicable

**N1**	18 (26.9)	23 (34.3)	20 (29.9)	6 (9.0)	0	61.2%	Not applicable


[Capital-letters (‘H’ and ‘N’) indicate H = main hospital physicians and N = non-main hospital physicians). Lower-case letters (‘a’, ‘b’, ‘c’ and ‘d’) and the digit (‘1’) abbreviate individual questions as listed in Table 4].

**Table 4:** Summary and Legend of Questions for Radiologists (R), radiologic technicians (T), referring physicians of the main hospital (H), and physicians of the non-main hospitals (N).


**Ra/Ta/Ha/Na**	Overall, how satisfied have you been with decentralized/modality-based radiology reporting (before system change)?

**Rb/Tb/Hb/Nb**	Overall, how satisfied have you been with centralized/subspecialized radiology reporting (after system change)?

**Rc/Hc/Nc**	How did you perceive the quality of radiology reports with decentralized/modality-based radiology reporting (before system change)?

**Rd/Hd/Nd**	How did you perceive the quality of radiology reports with centralized/subspecialized radiology reporting (after system change)?

**R1/T1**	How would you rate your subjective productivity/efficiency since changing from decentralized/modality-based to centralized/subspecialized radiology reporting (comparison between both evaluated systems of radiological reporting)?

**R2**	How did you perceive the quality of radiology reports since changing from decentralized/modality-based to centralized/subspecialized radiology reporting (comparison between both evaluated systems of radiological reporting)?

**R3**	How professionally confident are you in your assigned subspecialization since changing from decentralized/modality-based to centralized/subspecialized radiology reporting (comparison between both evaluated systems of radiological reporting)?

**Tc**	How would you rate the availability of residents (Radiology) with centralized/subspecialized radiology reporting (before system change)?

**Td**	How would you rate the availability of residents (Radiology) with centralized/subspecialized radiology reporting (after system change)?

**Te**	How would you rate the availability of senior radiologists with centralized/subspecialized radiology reporting (before system change)?

**Tf**	How would you rate the availability of senior radiologists with centralized/subspecialized radiology reporting (after system change)?

**H1/N1**	How would you rate the radiology turnaround time since changing from decentralized/modality-based to centralized/subspecialized radiology reporting (comparison between both evaluated systems of radiological reporting)?


[Capital-letters (‘R’, ‘T’, ‘H’ and ‘N’) indicate R = radiologists, T = radiologic technicians, H = main hospital clinicians and N = non-main hospital clinicians, lower-case letters (‘a’, ‘b’, ‘c’, ‘d’, ‘e’ and ‘f’) and the digits (‘1’, ‘2’ and ‘3’) abbreviate the individual questions as listed above].

Regarding the perceived quality of radiological reports (questions Rc/d, Hc/d, Nc/d), the surveyed groups of radiologists, referring physicians of the main hospital and referring physicians of the non-main hospitals had high agreement for all groups, ranging from 72,8% (Hc) to 98,1% (Rd). There were significant differences in the groups of radiologists (p = 0.013; ***Table 2***) with an agreement of 42/46 (91.3%) before the system change compared to 51/52 (98.1%) after the system change, and for the group of referring physicians of the main hospital (p = 0.001, ***Table 3***) with an agreement of 125/148 (72.8%) compared to 157/170 (92.4%). In the group of the non-main hospitals there was no significant difference noted with an agreement of 49/67 (73,1%) before the system change compared to 74/93 (79,5%) after the system change (p = 0.677, ***Table 3***).

With an agreement of 30/47 (63.8%) for the group of radiologists and 36/68 (53%) for the group of radiologic technicians, the two groups stated that their subjective productivity/efficiency (question R1/T1) had increased with centralized/subspecialized reporting when directly compared to decentralized/modality-based reporting (***Table 2***).

With an agreement of 33/48 (68.8%) the group of radiologists stated a better quality of their radiological reports (question R2) with centralized/subspecialized reporting when directly compared to decentralized/modality-based reporting (***Table 2***).

With an agreement of 29/44 (65.9%) the group of radiologists declared to feel more confident about the quality of their reports (question R3) with the centralized/subspecialized system when they directly compare both systems of radiological reporting (***Table 2***).

In the group of radiologic technicians, the agreement for the availability of radiology residents (questions Tc/d; ***Table 2***) were slightly lower after the system change [decentralized/modality-based system: 57/71 (80.3%); centralized/subspecialized system: 56/74 (75.7%)]. In contrary, agreement for the availability of board-certified radiologists (questions Te/f; ***Table 2***) were slightly higher after the system change [decentralized/modality-based system: 47/67 (70.1%); centralized/subspecialized system: 54/69 (78.2%)]. However, there were no significant differences in agreement for the availability of radiology residents or for the availability of board-certified radiologists (p = 0.87 respectively p = 0.277).

Regarding the radiology turnaround time (questions H1/N1; ***Table 3***), the surveyed groups of referring physicians of the main hospital and referring physicians of the non-main hospitals both stated an improved agreement of 104/144 (72.2%) respectively 41/67 (61.2%) when directly comparing the systems of centralized/subspecialized reporting and decentralized/modality-based reporting. When asked whether one of the two systems of radiological reporting was preferred, 45/47 (95.7%) of the consulted radiologists were in favor of the centralized/subspecialized system (***Figure 1***).

## Discussion

Literature research revealed that some of the variables of this study (i.e., satisfaction of radiologists, satisfaction of radiologic technicians, satisfaction of referring clinicians, availability of radiologists, and confidence of radiologists in their subspecialization) have not yet been investigated in terms of a system change from general to subspecialized radiology by other peer-reviewed published studies in English language.

As suggested by other studies [5,6,7,8], this survey also shows a significant difference in the perceived quality of radiological reports in the groups of radiologists (***Table 2***) and referring physicians of the main hospital (***Table 3***) in favor of subspecialized radiology. For the group of referring physicians of the main hospital, ***Table 3*** also shows a significant difference in agreement with overall satisfaction. In contrast, no significant differences were found for the group of referring physicians of the non-main hospitals. It is possible that referring physicians of larger centers may have different expectations of radiological reports and therefore require more precise reports, generally asking more specific or complex questions that a system change to subspecialized radiology may be able to answer better.

## Conclusion

Restructuring from decentralized/modality-based to centralized/subspecialized radiology resulted for the group of radiologists in a significant difference of the perceived quality of radiological reports. Furthermore, the consulted radiologists felt more confident when working in a subspecialization team. Out of all interviewed radiologists, 95.7% preferred the centralized/subspecialized system. The group of referring physicians of the main hospital stated a significant increased agreement regarding the items of overall satisfaction and the perceived quality of radiological reports. Specialized main hospital referring physicians value reports of specialized radiology, whereas less specialized, non-main hospital referring physicians did not experience any significant effect.
